# *The Organon* and Materia Medica Club of the Bay Cities of California

**Published:** 1896-02

**Authors:** W. E. Ledyard

**Affiliations:** Secretary


W. E. Ledyard, *Secretary.*

Reported by Eleanor F. Martin, M. D.

---

### *THE ORGANON* AND MATERIA MEDICA CLUB OF THE BAY CITIES OF CALIFORNIA.

The regular semi-monthly meeting was held at the office of Dr. J. E. Lilienthal, 1316 Van Ness Avenue, San Francisco, Friday evening, August 16th, 1895.

Members present: Drs. J. E. Lilienthal, J. M. Selfridge, A. McNeil, George H. Martin, E. W. Bradley, G. J. Auger, M. T. Wilson, and C. M. Selfridge.

The meeting was called to order at 8.45 by the President, Dr. J. M. Selfridge. As it was late for the meeting to begin, the reading of the minutes of the previous meeting was dispensed with and ordered to be read at the next meeting.

Dr. McNiel read a translation from the *Allgemeine Hom. Zeitung* of a case of cataract in a child ten years of age which was cured by one dose of *Sulphur*$^{500}$.

Dr. J. M. Selfridge read a paper entitled, "Infinitesimals in Nature." Dr. McNeil moved that the paper be sent to The Homœopathic Physician for publication, which was seconded by Dr. Wilson and carried.

DISCUSSION.

Dr. Chapman—I do not believe that in the DMM potency there is any material portion of the drug. I think that there is only the spirit force. I have seen results from this potency. In a case of mine that had taken Hood's Sarsaparilla for eczema of the right eye; was disfigured very badly; and had it many years; I gave Sulphur; then the DMM potency of the nosode, *eczema*, which cured.

Dr. Lilienthal—Dr. Chapman was not a member of our Club when we discussed the potency question. The DMM potency does not exist. It represents the 5 CM.

Dr. Chapman—I simply take what I read. I contend that we must drop matter and believe in a spirit force of drugs.

Dr. Auger—To my mind it is not necessary to go outside of materialization. Chemistry has taught us that atoms are units of matter. We need not go into spiritualism.

Dr. Chapman—I am not a spiritualist in the common sense of the word. I believe, however, that it is the spiritual man that is sick.

Dr. J. M. Selfridge—That is merely an assumption.

Dr. McNeil—If matter is infinitely divisible it is not necessary to bring in the spirit force to prove the efficacy of high potencies.

Dr. J. M. Selfridge—We used to think that we could not divide an atom, but now when appropriate means are used we can do so.

Dr. McNeil—That means infinite division.

Dr. Chapman—The doctrine of Hahnemann speaks of spiritual force.

Dr. J. M. Selfridge—We are not now living in Hahnemann's time. We cannot take Hahnemann's words as they appeared to him at that time. Science has made great advances since his time. It was held some ago that electricity was a fluid. It is now known to be a form of motion—rotary motion.

Dr. Chapman—Has science disproved anything that Hahnemann said?

Dr. McNeil—It has thrown new light on what he said.

Dr. J. M. Selfridge—While science has not controverted Hahnemann's conclusions, it explains them more clearly.

Dr. Martin—Dynamization is analogous to continuity of motion. If you drop a stone from the top of a house to the ground, the motion continues through the earth and on forever. What do you think thought is? It is analogous to the vibrations of the two tuning-forks spoken of by Dr. Selfridge, and is a transmission of molecular motion. Hypnotism is also a method of motion and is material.

Dr. Chapman—I positively deny that hypnotism is material. I have seen men in the army, before going into battle, say, "I am going to die this time," and they did. This must be something besides matter. One dark night, when riding in Nevada over a narrow trail, it came to me as if I heard a voice say, "get off that horse." I did so, and found a wire stretched across the trail, which would have thrown me down a precipice if I had gone on, and I would have been killed. I certainly do not call this material.

Dr. J. M. Selfridge—I am not denying that there is something outside of matter. It is not necessary, however, to suppose that we separate the spirit force of a drug from the drug itself and attach it to something else. If we can divide matter without limit, then a drug will go to the millionth and millionth of potencies. Remedies are all atoms, but differently combined. There is no philosophy in the fact that one spirit force makes *Arsenic* and another *Opium.* When a drug is divided up the material substance goes with it. Scientists do not go to the trouble to count these atoms. *There is no end to the division of matter.*

Dr. Chapman—Two substances may be identical as far as we know, yet we get entirely different results from their use.

Dr. J. M. Selfridge—There are no two peas alike. There is a different combination of molecules.

Dr. McNeil—Explanations of these matters are not necessary. As with an Indian owning a gun, he cannot explain what gunpowder is, but he knows that when it is put in his gun it will shoot and kill.

Dr. McNeil then read another translation from the *Allgemeine Hom. Zeitung.*

Dr. Chapman—When I was a young fellow I had bilious fever and an allopathic physician gave me Opium, one grain every three hours. I soon developed a very acute sense of smell. If there was a lemon in the room it had to be taken out. I could smell fruit-stands blocks away. I presume I had a proving of Opium.

The meeting then adjourned to meet at the office of Dr. J. M. Selfridge in Oakland, the first Friday in September, when the reading of *The Organon* would be commenced at Section 246.

